# Chemical Effect on Wellbore Instability of Nahr Umr Shale

**DOI:** 10.1155/2013/931034

**Published:** 2013-10-24

**Authors:** Baohua Yu, Chuanliang Yan, Zhen Nie

**Affiliations:** ^1^State Key Laboratory of Petroleum Resource and Prospecting, China University of Petroleum, Beijing 102249, China; ^2^Exploration & Production Research Institute, CNPC, Beijing 100011, China

## Abstract

Wellbore instability is one of the major problems that hamper the drilling speed in Halfaya Oilfield. Comprehensive analysis of geological and engineering data indicates that Halfaya Oilfield features fractured shale in the Nahr Umr Formation. Complex accidents such as wellbore collapse and sticking emerged frequently in this formation. Tests and theoretical analysis revealed that wellbore instability in the Halfaya Oilfield was influenced by chemical effect of fractured shale and the formation water with high ionic concentration. The influence of three types of drilling fluids on the rock mechanical properties of Nahr Umr Shale is tested, and time-dependent collapse pressure is calculated. Finally, we put forward engineering countermeasures for safety drilling in Halfaya Oilfield and point out that increasing the ionic concentration and improving the sealing capacity of the drilling fluid are the way to keep the wellbore stable.

## 1. Introduction

The Nahr Umr Shale Formation is found throughout the southern part of the Arabian Gulf and forms the cap rock to many major reservoirs in the region [1]. Major wellbore instability problems when drilling through this shale formation have often arisen not only in new wells but also in reentry wells, especially with the rise of water-based mud and stricter environmental control, making wellbore stability in this shale an extremely challenging operation for drilling/mud engineers.

The Halfaya Oilfield is in the south of Missan province in Iraq, which is 400 km south east of Baghdad, the capital of Iraq. Three horizontal wells in the Nahr Umr Formation of Halfaya Oilfield had been drilled. But, two wells of the three horizontal wells have sidetracking due to sticking, only one horizontal well is drilled successfully, so it illustrates the big effects of wellbore instability problems on the directional drilling in this oilfield.

## 2. Geological Character

The Halfaya Oilfield is located on the Arabian shelf, which is adjacent to the Zagros tectonic zone. The influence of Zagros tectonic movement is the extrusion to the Arabian shelf by the European plate (NNE-SSW). The propagation of the stress wave leads to a series of anticlines in the Arab shell. This extrusion stopped in Middle Miocene. The geological structure is a low dip anticline, in which the long axis is nearly perpendicular to the Zagros extrusion stress field [2–4]. The structure is above the Arabian shelf and is far away from the Zagros fault control zone, but the structure is still affected by Zagros tectonic movement, which makes the in situ stress complicated.

There is no large fault which could be recognized by seismic data. The anticline structure is also very smooth. The results show that the extrusion stress by the Zagros tectonic movement is not very strong, and the extrusion stress does not produce strong in situ deformation and destruction.

The lithologic characters in the Halfaya Oilfield from the top to the bottom is, respectively, the Tertiary Upper Fars Group, mainly sandy mudstone, about 1300 m thick; the Lower Fars Group, mainly anhydrite, salt rock, and shale deposit, about 500 m thick, being the regional cap rock; the Tertiary Kirkuk Group which is mainly sandstone and mudstone, about 300 m; from the Tertiary Jaddala group to the Nahr Umr group, mainly carbonatite and interlayers of thin marl, sandstone and shale. 

## 3. Drilling Problems

Three horizontal wells, N001H, N006H, and N002H, in the Nahr Umr Formation of Halfaya Oilfield had been drilled. The well distributions are both located in the structural long axis direction. The complicated drilling problems of these three wells are as follows.

When the first horizontal well (N001ST well) encountered the Nahr Umr layer, there were two sidetracking operations. The first sidetracking happened at 3941.26 m, the SLB screw stuck in the highly deviated interval and the directional tool dropped in the well. The fishing failed, which led to sidetracking. The second sidetracking happened at 4091.21 m, the Nahr Umr Shale collapsed and this led to sticking at 4087 m the treatment measures was ineffective and a sidetracking operation happened.

The second horizontal well (N006ST well) used the organic salt drilling fluid which has a strong inhibition. When drilling 3964 m, the Nahr Umr Shale collapsed and the treatment measure for the sticking failed, so sidetracking happened at 3800 m using the vertical well completion.

The third horizontal well N002H used saturated salt water drilling, but there were many sticks between 3660 m and 3895 m in the Nahr Umr Formation, and there were cavings at the shaking screen.

The two wells of the three horizontal wells have sidetracking due to sticking, only one horizontal well was drilled successfully, so it illustrates the big effects of wellbore instability problems in the oilfield. The wellbore instability has serious impact for oilfield drilling, and it restricts the exploration and development in the oil field. As the development wells are generally directional wells or horizontal wells, and the wellbore instability risk is great, wellbore stability analysis is needed in the Halfaya Oilfield. 

## 4. Wellbore Instability Mechanism

### 4.1. Character of the Instable Shale

**Figure 1:**
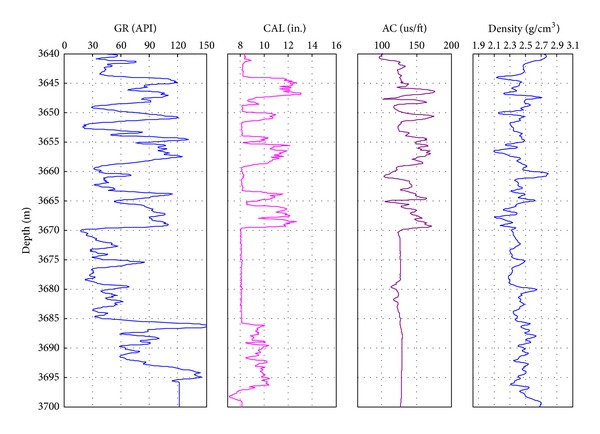
The comparison of the logging data in Nahr Umr Formation. GR: natural gamma logging; CAL: caliper logging; AC: acoustic transit time logging.

**Figure 2:**
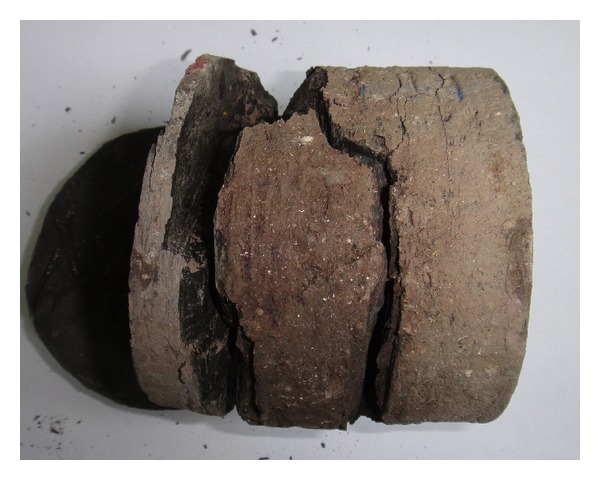
The core of Nahr Umr Shale.

**Figure 3:**
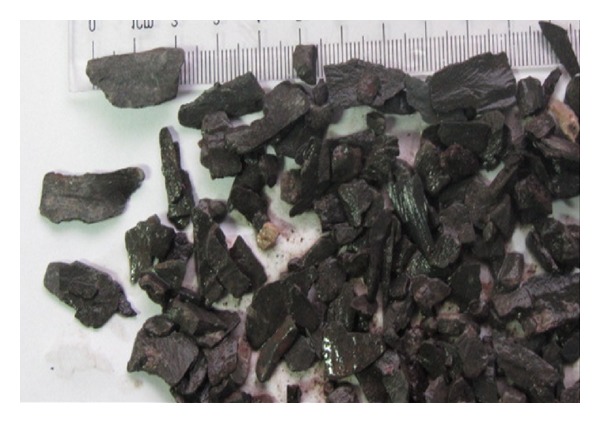
The shale cavings of the Nahr Umr Shale of N006H Well.

Figure 1 shows the logging data of the Nahr Umr Formation of N004 well. The GR logging shows that the formations are mainly sandstone and shale. The caliper logging shows that there are both stable and instable intervals. Compared to the GR logging data, the lithology of collapsing interval is shale and the sandstone interval is stable. According to the interval transit time logging data, the interval transit time of the Nahr Umr Shale is obviously higher than the adjacent sandstone interval. The density logging data of the shale are obviously lower than the adjacent sandstone interval. The reasons for this phenomenon are the rich internal microfractures, drilling fluid, and the filtrate seepage. This can be seen from the photo of the Nahr Umr Shale (Figure 2). In addition, the shape of cavings of the Nahr Umr Shale indicates that the shale is enriched in fractures (Figure 3). 

The main reason for wellbore instability in hard brittle shale with lots of fractures is as follows [5–11]. If the sealing capacity of the drilling fluid is not enough or the ionic concentration is not enough to balance the formation water ionic concentration, the drilling fluid and the filtrate would flow into the microfractures under the driving power from the fluid column pressure difference of drilling fluid and the ionic concentration difference. This would lead the friction coefficients of the fracture plane to decrease, the effective stresses around the wellbore to decrease, the formation around the wellbore to become loose, and the support of the drilling fluid column to the wellbore wall decrease. Thus the formation fluid will flow into the wellbore. During the reaming and back reaming, the disturbance of the rigs to the loose formation will lead to wellbore instability.

### 4.2. Wellbore Instability Analysis

In order to solve the wellbore instability of the fractured and hard-brittle shale in Nahr Umr Formation, the drilling fluid property and engineering countermeasures should be taken into account. We analyze the wellbore instability reasons combined with the drilling engineering measures of three horizontal wells in the Nahr Umr Formation.

When using rational engineering measures, the drilling fluid property decides the wellbore stability during drilling to a large extent. Table 1 shows the drilling fluid property used in these three horizontal wells. These three wells used three different types of drilling fluid. The following can be concluded from the drilling fluid property parameters in the table.

(1) Based on the mud rheological parameters, for the formation with good completeness, the rheological parameters of these three wells are similar and could meet the engineering requirement. But, for the fractured shale formation, the rheological parameters of these three wells are different. Compared to the other two wells, the drilling fluid of N001H well has a low viscosity, which is bad for carrying the cuttings and cavings. In addition, the drilling fluid with low viscosity will easily flow into the formation under the pressure difference. Therefore, the rheological parameters of drilling fluid of N002H well benefit wellbore stability. Usually increasing the drilling fluid viscosity is of benefit for fracture formation.

(2) Based on the drilling fluid filter loss, the filter losses of these three wells are similar. Because the filter loss is measured by the experimental instrument in the laboratory, the results cannot reflect the real formation situation and it is only a reference index.

(3) Based on the drilling fluid ionic concentration, although there are not ionic concentration parameters of N001H well drilling fluid in the daily drilling report, according to the drilling fluid description provided by the drilling fluid service provider, the drilling fluid ionic concentrations of this well could indicate that the ionic concentration of KCL Polymer drilling fluid used in the N001H well is between the concentrations of the N002H well and N006H well; the ionic concentration of the N002H well is the highest the ionic concentration of the N006H well is the lowest. When the hole is opened, the ionic concentration difference of the drilling fluid and formation water is the main driving force that drives the free water in the drilling fluid into the formation. Commonly, the high ionic concentration of drilling fluid is of benefit to prevent the free water in the drilling fluid from flowing into the formation. If the free water in the drilling fluid flows into the formation, the formation will be hydrated, and the formation strength will be decreased, so as to lead to wellbore periodic collapsing.  Table 2 shows the formation water property of Halfaya Oilfield. The results show that the formation water has an extremely high ionic concentration, which needs a high ionic concentration for drilling fluid to balance it.

### 4.3. Shale Hydration

According to the formation character, the Nahr Umr Shale is abundant in microfractures; the formation is broken, and the drilling fluid can easily flow into the micro fracture plane, which leads to the change of formation strength. In order to prevent wellbore instability, the drilling fluid property should be improved. The influence of drilling fluid on the wellbore stability is analyzed from the mineral composition, the drilling fluid consistency, and the influence of the drilling fluid on formation strength.

Tables 3 and 4 illustrate the minerals and clay minerals composition and content of the Nahr Umr Shale, respectively. The test results in the tables show that the Nahr Umr Shale mainly consists of quartz and clay, especially quartz, which exceeds 48.5%. For shale Formation, the higher the quartz, the higher the brittleness; at the same time, the content of the clay minerals of the shale belongs to medium and little high level. The clay minerals mainly consist of illite/smectite and kaolinite, and the content of smectite is low. The type of the clay mineral indicates that the shale is very brittle. In addition, the kaolinite is a stable clay mineral, and the hydration of the illite/smectite is also feeble. The type and content of the clay minerals both indicate that Nahr Umr Shale Formation is a hard and brittle formation which is hard to hydrate.

According to research experience, if the drilling fluid inhibition is good enough, a formation like the Nahr Umr Shale is impossible to hydrate without expansion collapsing. Therefore, we evaluate the rejection capacity of three drilling fluid systems which are used in the Halfaya Oilfield. The three drilling fluids are organic salt drilling fluid, Gel-polymer drilling fluid, and KCl-polymer drilling fluid.

The densities of these three types of drilling fluid are all 1.33 g/cm^3^; then we measured the cuttings recovery (The 40 g cuttings with 3.2~2.0 mm diameter injected to the 350 mL fluid. Roll 16 h in a set temperature. Then filter the cuttings through a sieve. Dry and weigh cuttings to calculate the cuttings recovery.) and swelling ratio of these three drilling fluids. The results are shown in Table 5. The results show that the cuttings recoveries of these three types of drilling fluid are both higher than 95% for the Nahr Umr Shale; although the swelling ratios are different. These results show that the inhibitive capacity of the drilling fluid is good [12]; on the other hand, the results indicate that the formation hydration is feeble. The inhibitive capacity of the drilling fluid is not the main reason for the wellbore instability of the Nahr Umr Shale.

**Figure 4:**
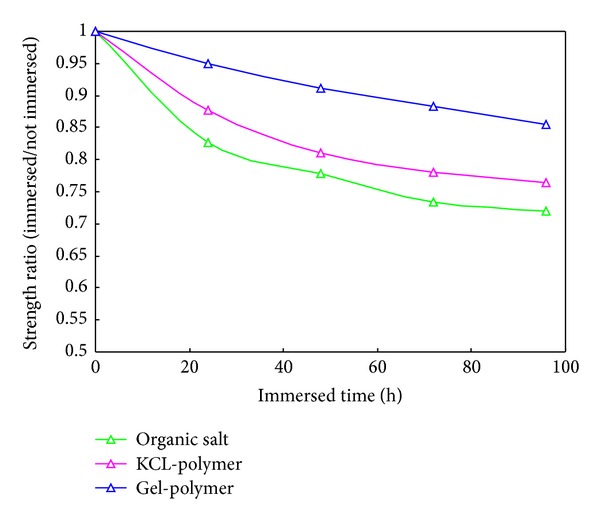
Comparison of the shale strength decrease after immersing.

In order to analyze the influence of the drilling fluid on the wellbore stability of the Nahr Umr Shale, experimental studies were carried on the influence of drilling fluid on the rock mechanical property. We tested the shale strength of Nahr Umr Shale after immersing it in different kinds of drilling fluids.  Table 6 shows the uniaxial compressive strength (UCS, MPa) results from the test.  Figure 4 illustrates the comparison of the strength variation rule versus the time after immersing in different kinds of drilling fluid.

Figure 4 shows that the shale UCS decreases greatly after immersing it in the organic salt drilling fluid, the next is the KCL-polymer drilling fluid; the strength in the Gel-polymer drilling fluid changed a little. Therefore, the Gel-polymer drilling fluid benefits the wellbore stability of the Nahr Umr Shale.

Under the drive force of the ionic concentration difference, the free water in the drilling fluid which flows into the formation would decrease the rock strength, which is the main reason for the collapse in Nahr Umr Shale; in addition, as the formation is extremely hard and brittle and the fractures are rich internally, if the drilling fluid sealing capacity is not good enough, the drilling fluid and filtrate would flow into the rock along the microfracture under the difference of the drilling fluid column pressure and pore pressure, so as to weaken the formation strength and lead to wellbore collapse. So, the increasing of the ionic concentration of the drilling fluid and enhancing the drilling fluid sealing capacity is the key to the wellbore stability of the Nahr Umr Shale.

## 5. Time-Dependent Collapse Pressure

According to mechanical concepts, the main reason for borehole collapse is caused by shear failure for the reason that stresses loaded on rock around the borehole exceed the rock strength, as a result of lower mud column pressure. Now, brittle formation collapse will generate and the borehole will enlarge; for plastic formation, plastic deformation be will generated and borehole shrinkage will be encountered.

Generally, borehole collapse takes place in the minimum horizontal stress direction, *θ* = *π*/2 or 3*π*/2 [12]; the borehole stress on minimum horizontal stress direction [13–20] is as follows:

(1) $$\begin{array}{c} \sigma_{r}=P-\delta \phi \left(P-P_{p}\right),\\ \sigma_{\theta }=3\sigma_{H}-\sigma_{h}-P+\delta \left[\frac{\alpha \left(1-2\nu \right)}{1-\nu }-\phi \right]\left(P-P_{p}\right),\\ \sigma_{z}=\sigma_{v}+2\nu \left(\sigma_{H}-\sigma_{h}\right)+\delta \left[\frac{\alpha \left(1-2\nu \right)}{1-\nu }-\phi \right]\left(P-P_{p}\right). \end{array}$$

Assume that safe coefficient FS [21] is as follows:

(2) $$\begin{array}{c} \text{FS}=\frac{\sigma_{n}tg\varphi +C}{\tau }. \end{array}$$

And let

(3) $$\begin{array}{c} M=1+\left(\text{FS}-1\right)\cos^{2}\varphi . \end{array}$$

Replace normal stress *σ*_*n*_ as principal stress *σ*_1_ and *σ*_3_:

(4) $$\begin{array}{c} \sigma_{n}=\frac{\sigma_{1}+\sigma_{3}}{2}-\frac{\sigma_{1}-\sigma_{3}}{2}\sin\varphi -\alpha P_{p}. \end{array}$$

Rewrite Mohr-Coulomb criterion [21]:

(5) $$\begin{array}{c} M\left(\sigma_{1}-\sigma_{3}\right)-\sin\varphi \left(\sigma_{1}+\sigma_{3}-2\alpha P_{p}\right)-2C\cos\varphi =0. \end{array}$$

Based on different borehole stress conditions, borehole collapsing pressure expresses as a different form. When the bearing condition is *σ*_*θ*_ > *σ*_*z*_ > *σ*_*r*_, maximum and minimum stress separately are *σ*_1_ = *σ*_*θ*_, *σ*_3_ = *σ*_*r*_, and make *k* = (*α*(1 − 2*ν*)/(1 − *ν*)) − *ϕ*; under mud penetrating borehole face, the borehole collapsing pressure model is

(6) Pcr=(2Ccos⁡ϕ+(sinϕ−M)(3σH−σh) +[δMk+sinϕ(2δf−δk−2k)]Pp)×(δksinϕ−M(2+δk−2δf))−1.

For the hard-brittle shale of Nahr Umr Formation, according to the study results and experimental results, the influence of the drilling fluid immersion on the mechanical property mainly reflects in the decrease of the compressive strength as the immersing time increase. 

**Figure 5:**
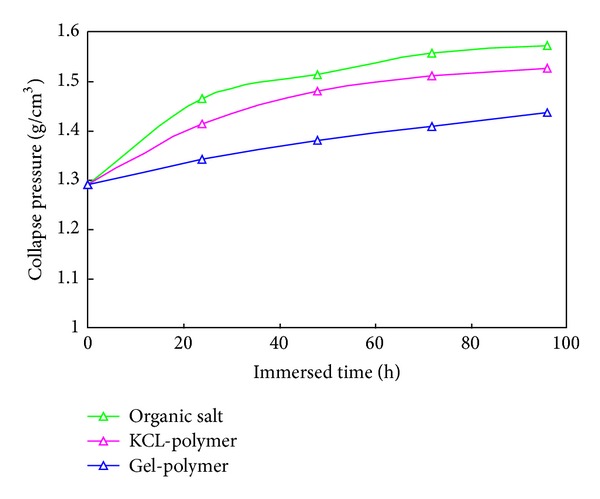
Time-dependent collapse pressure of Nahr Umr Shale.

Figure 5 illustrates the variation of collapse pressure versus the hole opening time for Nahr Umr Shale. The collapse pressure would increase as the formation strength decreases; the increasing speed decreases gradually. The increasing rate of Gel-polymer drilling fluid is the lowest; in a certain drilling fluid density it can keep the wellbore stability for the longest time. The increasing of the mud density could only keep the wellbore stability in limited time. If the property of the drilling fluid cannot be improved, increasing the mud density would force the drilling fluid to flow into the formation and make the wellbore unstable.

## 6. Countermeasures Dealing with Wellbore Instability

In order to prevent the wellbore instability of Nahr Umr Shale, we come up with the following drilling technology countermeasures and suggestions.

- Depending only on the drilling fluid density cannot solve the wellbore stability of the shale formation which is full of fractures [22, 23]. If the drilling density is too high, the pore pressure would increase and the effective stresses around the wellbore decrease, and this would cause a larger damaged scale. Decreasing the drilling fluid filter loss and improving the drilling fluid rheological property would benefit wellbore stability.
- Commonly, the larger the inclination, the more possible the wellbore instability. But for the laminar fracture formation, decreasing the angle of the wellbore axial line with the bedding normal direction is of benefit for the wellbore stability.
- The influences of the swabbing pressure and surge pressure should be taken into consideration when evaluating wellbore stability; the simplified bottom hole assembly (BHA) could prevent large swabbing pressure and surge pressure and then prevent sticking.
- The hydraulic jetting is not suitable, because the high pressure hydraulic jetting would produce water wedge effect in the progress of the drilling seepage. The big diameter jet or no-jet are welcomed.
- Avoiding the intense change of the dogleg or the well track so as to prevent big drill string acting force to the wellbore wall.
- Optimizing the hydraulic parameters so as to ensure the cuttings could be carried out of the wellbore timely. For some situations, wellbore collapse can not be prevented, so carrying out the cuttings in a timely way could decrease the downhole complicated time. Increasing the drilling rate could decrease the exposed time of the shale formation, which is useful for the wellbore stability.
- The formation water has an extremely high ionic concentration, so keep a high ionic concentration for the drilling fluid to balance it.

## 7. Conclusions

Under the function of the ionic concentration difference, the free water in the drilling fluid which flows into the formation will decrease the rock compressive strength, which is the main reason for the collapse in Nahr Umr Shale; in addition, as the formation is extremely hard and brittle and the fractures are internally rich, if the drilling fluid sealing capacity is not good enough, the drilling fluid and filtrate will flow into the rock along the micro fracture surface under the difference of the drilling fluid column pressure and pore pressure, so as to weaken the formation strength and lead to wellbore collapse. So, increasing the ionic concentration of the drilling fluid to enhance the drilling fluid sealing capacity is the key point to the wellbore stability of the Nahr Umr Shale Formation.

The collapse pressure will increase as the formation strength decreases after drilling; the increasing speed decreases gradually. The increasing rate of Gel-polymer drilling fluid is the lowest; in a certain drilling fluid density it can keep the wellbore stable for the longest time. The increase of the mud density could only keep the wellbore stability for a limited time. Improving the property of the drilling fluid is the basis for keeping the wellbore stable.

## Figures and Tables

**Table 1:** The drilling fluid properties of three horizontal wells of Nahr Umr Formation.

	Well no.			
	N001H well-Hole 1	N001H well-Hole 2	N002H well	N006H well
Mud type	KCL-polymer	KCL-polymer	Salt saturated	BH-WEI
Density (g/cm^3^)	1.25	1.25	1.28	1.28
Viscosity (s)	51	53	78	65
Plastic viscosity (cp)	26	27	41	39
Y.P (lb/100 ft^2^)	24	26	31	29
Gel strength 10′′/10′ (lb/100 ft^2^)	5/8	5/14	7/9	5/7
API filtrate (mL)	3.2	3.4	3.0	3
Mud cake (mm)	0.3	0.3	0.3	0.5
PH	9.5	9	9	8.5
Solid (%)	13	11	13	17
Sand (%)	0.3	0.3	0.2	0.3
Bentonite content (g/L)	27	26	38	
Potassium (mg/L)			27000	
Chloride (mg/L)			55000	11520
Ca^+^ (mg/L)			200	

**Table 2:** The formation fluid properties of Halfaya oilfield.

	Unit	Nahr Umr
Water type		CaCl_2_
PH		6.3
Specific gravity (15.56°C)	sg	1.121
Resistivity (25°C)	ohm·m	0.068
Total salinity	ppm	166661
Total hardness	mg/L	16562
Na^+^	mg/L	60015
Ca^2+^	mg/L	8681
Mg^2+^	mg/L	993
Fe^2+^	mg/L	74
Ba^2+^	mg/L	1
K^+^	mg/L	716
Sr^2+^	mg/L	356
Cl^−^	mg/L	107098
SO_4_^2−^	mg/L	874
HCO_3_^−^	mg/L	7263
CO_3_^2−^	mg/L	0
OH^−^	mg/L	0

**Table 3:** Mineral composition and content of the Nahr Umr Shale.

Depth	Mineral content (%)								
	Quartz	Potassium feldspar	Soda feldspar	Anorthose	Calcite	Dolomite	Iron pyrite	Hematite	TCCM
3645.10	51.7	0.8		0.2				2.7	44.6
3649.83	60.8	1.2			0.4		4.7		32.9
3666.00	48.5	1.9		0.3	1.4		4.5		43.4

**Table 4:** Clay mineral composition and content of the Nahr Umr Shale.

Depth	Clay mineral content (%)						Interbed ratio (% S)	
	S	I/S	It	Kao	C	C/S	I/S	C/S
3645.10		34	7	48	11		14	
3649.83		33	3	40	24		11	
3666.00		44	7	49			21	

**Table 5:** Swelling ratio and recovery of the Nahr Umr Shale.

	Organic salt	KCL-polymer	Gel-polymer
Recovery Rate (%)	95	96	97
Swelling Ratio (%)	24	36	22

**Table 6:** Experimental results of shale UCS after immersing in drilling fluid.

Drilling fluid type	Organic salt	KCL-polymer	Gel-polymer
UCS without immersing (MPa)	48.62	51.09	47.22
UCS with immersing of 24 h (MPa)	40.16	44.8	44.8
UCS with immersing of 48 h (MPa)	37.81	41.41	43.02
UCS with immersing of 72 h (MPa)	35.64	39.82	41.69
UCS with immersing of 96 h (MPa)	34.96	39	40.33
